# Low self-reported sports activity before stroke predicts poor one-year-functional outcome after first-ever ischemic stroke in a population-based stroke register

**DOI:** 10.1186/s12883-018-1189-y

**Published:** 2018-11-03

**Authors:** Christian Urbanek, Viola Gokel, Anton Safer, Heiko Becher, Armin J. Grau, Florian Buggle, Frederick Palm

**Affiliations:** 1 Department of Neurology, University of Heidelberg, Städtisches Klinikum Ludwigshafen am Rhein, Bremserstr. 79, 67063 Ludwigshafen, Germany; 2 University of Mannheim, Department of Dermatology, Theodor-Kutzer-Ufer 1-3, Mannheim, 68167 Germany; 3 University of Heidelberg, Institute of Public Health, Im Neuenheimer Feld 324, Heidelberg, Germany; 4 Institute of Medical Biometry and Epidemiology, University Medical Center Hamburg-Eppendorf, Hamburg, Germany

**Keywords:** Stroke, Cerebral infarction, Outcome, Physical activity, Predictors, Risk factors

## Abstract

**Background:**

Physical activity (PA) is associated with lower risk of stroke. We tested the hypothesis that lack of pre-stroke PA is an independent predictor of poor outcome after first-ever ischemic stroke.

**Methods:**

We assessed recent self-reported PA and other potential predictors for loss of functional independence - modified Rankin Scale (mRS) > 2 - one year after first-ever ischemic stroke in 1370 patients registered between 2006 and 2010 in the Ludwigshafen Stroke Study, a population-based stroke registry.

**Results:**

After 1 year, 717 (52.3%) of patients lost their independence including 251 patients (18.3%) who had died. In multivariate logistic regression analysis lack of regular PA prior to stroke (Odds Ratio (OR) 1.7, Confidence Interval (CI) 1.1–2.5), independently predicted poor outcome together with higher age (65–74: OR 1.7; CI 1.1–2.8, 75–84 years: OR 3.3; CI 2.1–5.3; ≥85 years OR 14.5; CI 7.4–28.5), female sex (OR 1.5; CI 1.1–2.1), diabetes mellitus (OR 1.8; CI 1.3–2.5), stroke severity (OR 1.2; CI 1.1–1.2), probable atherothrombotic stroke etiology (OR 1.8; CI 1.1–2.8) and high leukocyte count (> 9.000/mm^3^; OR 1.4; CI 1.0–1.9) at admission. Subclassifying unknown stroke etiology, embolic stroke of unknown source (ESUS; *n* = 40, OR 2.2; CI 0.9–5.5) tended to be associated with loss of independence.

**Conclusion:**

In addition to previously reported factors, lack of PA prior to stroke as potential indicator of worse physical condition, high leukocyte count at admission as indicator of the inflammatory response and probable atherothrombotic stroke etiology might be independent predictors for non-functional independence in first-ever ischemic stroke.

## Background

A high proportion of stroke survivors worldwide require assistance or are fully dependent on caregivers for activities of daily living after stroke [1]. Improved individualized therapy in acute ischemic stroke care, preemptive therapy of risk factors or changes in lifestyle prior to stroke may modify ischemic stroke (IS) outcome. Prediction of functional outcome in patients with IS can support clinicians to improve effective stroke care, anticipate discharge planning and support patients and family to develop realistic expectations for long-term care provision.

Clinical rating or imaging - based scoring systems like ASTRAL, DRAGON or SEDAN have been published to predict loss of functional independence after IS [2–5]. Age, initial stroke severity, onset to admission time, range of visual fields, level of consciousness, glucose and concentrations of serum neutrophil markers were some predictors for losing functional independence in these studies [6–11]. However, prognostic models had only minor impact on clinical practice. The majority of these scores were based on retrospective analysis of cases from hospital-based data [3, 12–18]. Few studies have systematically evaluated multiple factors in prospective and unselected data series of consecutive ischemic stroke patients [19–23].

Physical activity (PA) activity before stroke as measured by self-report adds to the risk of poor outcome.

Epidemiologic studies have consistently suggested an association between PA and the risk of stroke [24–27]. PA is recommended to reduce the risk of first-ever and possibly the risk of recurrent stroke [24, 28–30]. Low PA may lower the individual capacity to cope with the metabolic and other stressful sequelae after cerebral ischemia.

The aim of this study was to identify predictors of one-year-functional outcome in patients with first-ever ischemic stroke, using five-year case series data from a prospective, population-based stroke registry. In particular, we tested the hypothesis that lack of self-reported recent PA increases risk of poor functional outcome after IS. Besides the well-established risk-factors, inflammatory parameters such as leukocyte count and fibrinogen were added to our analysis as previous studies showed an effect of a high inflammatory response on stroke outcome [31, 32].

## Methods

The “Ludwigshafen Stroke Study” (LuSSt) is a prospective population-based stroke register in Ludwigshafen at Rhine in Germany, that started on January 1st, 2006 [33].

In order to achieve complete case ascertainment, multiple overlapping methods of patient identification were used as described previously [33]. Case ascertainment of hospitalized patients was ensured by collaboration with all hospitals in the city of Ludwigshafen and hospitals in the region. To identify all non-hospitalized stroke patients, general practitioners, specialists in internal medicine, and neurologists practicing in Ludwigshafen were informed about the register before study initiation and were contacted together with nursing and residential homes. All patients treated at “Klinikum Ludwigshafen” were examined by a member of the study team, including an interview based on a structured questionnaire as described previously [34]. We intended to keep the questions as simple as possible for interviews in acutely ill patients. All patients who have been treated outside “Klinikum Ludwigshafen” and gave informed consent, were examined by a member of the study team. In the other patients data were obtained by the attending physician and transmitted to the study center in pseudonymised form. In all patients with informed consent follow-up investigations were performed by telephone 1, 3 and 12 months after stroke utilizing a standardized questionnaire. If patients were unable to provide informations, a next-of-kin was interviewed. In patients without informed consent, or without response to multiple telephone and letter contact attempts, survival and death information was obtained by the population registration authority. LuSSt has been approved by the ethics committee of the Landesärztekammer Rhineland-Palatinate and by the data protection commissioner of Rhineland-Palatinate.

Stroke was defined according to the definition of the World Health Organization (WHO) [35]. Stroke subtype classification was based on the results of brain imaging, discriminating between IS, intracerebral hemorrhage (ICH), and subarachnoid hemorrhage (SAH). In case brain imaging was unavailable stroke type was defined as undetermined. The present analysis comprises only patients with first-ever ischemic stroke up to December 31st, 2010. Patients with a first ischemic stroke and a history of transient ischemic attack (TIA) were coded as first-ever ischemic stroke according to comparable population-based stroke registries [36]. Patients with recurrent stroke, TIA, SAH and ICH were excluded for present analysis.

### Outcome parameters and risk factors

Stroke severity was determined at hospital admission using the National Institute of Health Stroke Scale (NIHSS) [37]. In order to assess functional status prior to stroke and functional outcome after first-ever ischemic stroke, modified Rankin Scale (mRS) was used [38, 39]. MRS is a 7-point scale ranging from 0 (no symptoms) to 6 (death). A score of 2 or less indicates functional independence [40]. Loss of independence in daily life was defined as a mRS > 2 summarizing patients that had survived first 12 months after stroke with significant disabilities, and deceased ones. Cardiovascular risk factors were defined according to current guidelines as described previously [34, 41, 42]. Definitions have already been described earlier [34, 43]. In brief, hypertension was diagnosed if the patient was on antihypertensive medication on admission, if hypertension had been diagnosed before by a physician or if blood pressure was > 140/90 mmHg in two or more measurements > 3 days after stroke. Diabetes mellitus was defined in subjects with fasting blood glucose level above 125 mg/dl in venous blood, present anti-diabetic medication at hospital or known diagnosis of diabetes mellitus. Diagnosis of atrial fibrillation (AF) has been made if permanent or paroxysmal AF was present on ECG or long-term monitoring and additionally, in case of a history of this diagnosis. All in-patients with cholesterol-lowering medication, fasting cholesterol levels > 200 mg/dl or LDL-cholesterol > 140 mg/dl lead to diagnosis of Hypercholesterolemia. We defined current smoking as present daily usage of any kind of tobacco (at least one cigarette, cigar, cigarillo or pipe). We classified history of smoking as smoking for any period of at least 6 months. Patients with previous angina pectoris, myocardial infarction, coronary stenting or coronary artery bypass were selected as subjects with coronary artery disease (CAD) [34]. Patients with medical history of peripheral artery disease (PAD), arterial bypass surgery, stenting vessels of lower limbs and patients with present intermittent claudication or history of intermittent claudication of vascular origin were diagnosed PAD. Alcohol consumption as measured by self-report was coded if > 1 drink per week was consumed on a regular base. Another selection criteria was consumption of alcohol in the past.

We used definition of the German Olympic Sports Association for PA as formerly described: PA as any leisure-time motor activity that had its aim in itself or was performed for no other purpose than to improve or maintain physical fitness [43]. Therefore, all activities such as walking were defined as PA and had been included. However, PA during work, PA on the way to or from employment or activities like gardening were not considered. All subjects were asked whether they had regularly performed sports during the months before stroke [30, 43]. Regular PA was acknowledged as such activity at least once a week.

C-Reactive Protein (CRP) [particle-enhanced immunoturbidimetric assay CRPL3 (cobas®)], fibrinogen (Clauss method on IL Coagulation Systems, Instrumentation Laboratory) and leukocyte count (XE analyserXE-2100; Sysmex) were determined < 48 h after admission.

#### Medical treatment

Thrombolysis was defined as intravenous application of recombined tissue plasmin activator (rt-PA). During the early years of LuSSt, mechanical recanalisation was not a standard in acute stroke therapy, and therefore not captured in the database. Antiplatetlet treatment included usage of one or more of these drugs: acetylsalicyl acid, clopidogrel, dipyridamol with acetylsalicylic acid and inhibitors of glycopeptide IIb/IIIa.

#### Classification of stroke etiology

We used a modification of the TOAST (Trial of Org 10,172 in Acute Stroke Treatment) criteria to define etiological subtypes of ischemic stroke [44]. Stroke due to large-artery atherosclerosis, cardioembolism, small-artery occlusion, stroke of other determined cause and stroke of undetermined etiology (except such from two or more competing etiologies) were diagnosed according to the TOAST criteria. In addition, we diagnosed ‘probable atherothrombotic stroke’ in such patients with stenosis < 50% diameter reduction on duplex sonography, CT-, MR- or digital subtraction angiography and additional brain infarction(s) > 1.5 cm in the absence of any source of cardioembolism. This category is comparable to “atherothrombotic stroke” in the PERFORM study [45]. In patients with more than one potential cause for stroke, etiology was assigned to the most likely causative mechanism according to the SSS-TOAST classification [46]. Patients with stroke of unknown etiology were analyzed retrospectively, specifically using embolic strokes of undetermined source criteria (ESUS) and reclassified in cryptogenic ESUS, cryptogenic NON-ESUS or stroke of undetermined source (incomplete work-up or concurrent stroke). Classification was performed by experienced neurologists of the study team [47]. Controversial diagnoses were discussed and agreed in study meetings.

## Statistical methods

For univariate analyses χ^2^-test, t-test with and without log-transformation and the Wilcoxon test were used as appropriate. For multivariate analysis, logistic regression was used. Variables being significant in univariate analyses were included in multivariable logistic regression analysis using the backward elimination procedure. To analyse the influence of early deaths after stroke, we compared our full dataset (Model A) with results after excluding patients who died early within the first 7 days after stroke (Model B). In further analysis, patients with “first ever ischemic stroke of unknown cause” were divided into three groups: “cryptogenic ESUS”, “cryptogenic NON-ESUS” and “stroke of undetermined source” (stroke with incomplete work-up or concurrent stroke) (Model C).

All data were analysed using SAS 9.4 software (SAS Institute, North Carolina). All tests were performed for two-sided testing. Level of significance was set to α = 0.05 for all tests.

## Results

Between January 1st, 2006 and December 31st, 2010, 1547 cases of first-ever ischemic stroke were registered in LuSSt. One-year follow-up information was available for 1370 subjects (88.6%, 677 women and 693 men). Information on mRS prior to stroke was available in 930 patients among whom 63 patients (4.6%) had a mRS > 2. No significant differences between patients with and without follow-up existed regarding age, sex and NIHSS at admission (*p* > 0.1, respectively). Among the 1370 patients, 717 patients (52.3%) had poor outcome with loss of functional independence including 251 (18.3%) patients who had died within the first year.

Clinical characteristics of all patients by one-year functional outcome are shown in Table 1. In univariate analysis, female sex, higher age, higher NIHSS score at admission, stroke etiology, higher leukocyte count, higher fibrinogen level, antiplatelet drugs before stroke, intravenous thrombolysis, arterial hypertension, AF, CAD, hypercholesterolemia, diabetes mellitus, PAD, smoking status, alcohol consumption, regular PA prior to stroke and mRS > 2 at discharge were associated with one-year poor functional outcome or loss of independence. Among 117 patients with ischemic stroke of unknown etiology, stroke workup was incomplete in 39 (33.3%) patients, cryptogenic stroke was diagnosed in 60 (51.3%) patients and more than one possible etiology for ischemic stroke was evident in 18 patients (15.4%). Among patients with cryptogenic stroke, ESUS was diagnosed in 40 (66.7%) patients.

**Table 1:** Baseline characteristics, clinical characteristics and cerebrovascular risk factors by functional-outcome in 1370 patients with first-ever ischemic stroke – *univariate analysis*

Predictor variable (number of missing observations total)	One-year functional outcome			
	N (%) ^&^Median (lower-upper quartile)			*p* value
	Total (*n* = 1370)	mRS ≤ 2 (*n* = 653)	mRS > 2 (*n* = 717)	
Sex (0)				< 0.01
Men	693 (50.6)	385 (59)	308 (43)	
Women	677 (49.4)	268 (41)	409 (57)	
Age (mean ± SD;years) (0)	71.6; ±13	66.1; ±12.3	76.6; ±11.4	< 0.01
Vascular risk factors				
Arterial hypertension (11)				< 0.01
Yes	1195 (87.9)	549 (84.6)	646 (91)	
No	164 (12.1)	100 (15.4)	64 (9)	
Atrial fibrillation (36)				< 0.01
Yes	391 (29.3)	112 (17.6)	279 (39.9)	
No	943 (70.7)	523 (82.4)	420 (60.1)	
Coronary heart disease (46)				< 0.01
Yes	304 (23)	125 (19.7)	179 (25.9)	
No	1020 (77)	509 (80.3)	511 (74.1)	
Hypercholesterolemia (36)				< 0.01
Yes	884 (66.3)	465 (72.5)	419 (60.5)	
No	450 (33.7)	176 (27.5)	274 (39.5)	
Diabetes (20)				< 0.01
Yes	422 (31.3)	167 (25.9)	255 (36.1)	
No	928 (68.7)	477 (74.1)	451 (63.9)	
Peripheral arterial disease (51)				< 0.01
Yes	132 (10)	46 (7.3)	86 (12.6)	
No	1187 (90)	588 (92.7)	599 (87.4)	
Smoking (0)				< 0.01
Yes, actually	305 (22.3)	184 (28.2)	121 (16.9)	
Yes, in the past	394 (28.8)	196 (30)	198 (27.6)	
No	520 (38)	224 (34.3)	296 (41.3)	
Unknown	151 (11)	49 (7.5)	102 (14.2)	
Consumption of alcohol^a^ (80)				< 0.01
Yes, actually	504 (39.1)	277 (44.3)	227 (34.1)	
Yes, in the past	38 (2.9)	17 (2.7)	21 (3.2)	
No	665 (51.6)	302 (48.3)	363 (54.6)	
Unknown	83 (6.4)	29 (4.7)	54 (8.1)	
Regular physical activity (0)				< 0.01
Yes	266 (19.4)	187 (28.6)	79 (11)	
No	1104 (80.6)	466 (71.4)	638 (89)	
Clinical characteristics on admission				
NIHSS (33)^b^	3 (2–6)	2 (1–4)	5 (3–11)	< 0.01
TOAST (0)				< 0.01
Probable atherothrombotic	208 (15.2)	98 (15)	110 (15.3)	
Cardioembolic	422 (30.8)	143 (21.9)	279 (39)	
Large-artery atherosclerosis	192 (14)	85 (13)	107 (14.9)	
Small-artery occlusion	369 (27)	237 (36.3)	132 (18.4)	
Other determined	62 (4.5)	28 (4.3)	34 (4.7)	
Unknown	117 (8.5)	62 (9.5)	55 (7.7)	
Leukocytes (47)^b^	8.3 (6.8–10.2)	7.9 (6.6–9.7)	8.6 (7.0–10.7)	< 0.01
Fibrinogen (86)^b^	370 (318–434)	356 (309–412)	386 (329–455)	< 0.01
Antiplatlet drugs (0)				< 0.01
Yes	480 (35)	197 (30.2)	283 (39.5)	
No	890 (65)	456 (69.8)	434 (60.5)	
Lysis therapy (0)				< 0.01
Yes	124 (9.1)	37 (5.7)	87 (12.1)	
No	1246 (90.9)	616 (94.3)	630 (87.9)	
Clinical characteristics at discharge				
NIHSS (109)^b^	2 (1–4)	1 (0–2)	3 (1–7)	< 0.01
mRS (94)				
≤ 2 (0–2)	838 (65.7)	573 (92)	265 (40.6)	< 0.01
> 2 (3–6)	438 (34.3)	50 (8)	388 (59.4)	

Comparisons by X^2^-test, Student’s t-test with and without log-transformation and Wilcoxon test as appropriate

*mRS* modified Rankin Scale, *N* number, *NIHSS* National Institutes of Health Stroke Scale, *SD* standard deviation, *TOAST* Trial of ORG 10172 in Acute Stroke Treatment; ^a^ > 1 drink per week; ^b^ Quartiles

In multivariate analysis, lack of regular PA prior to stroke (OR 1.7; CI 1.1–2.5) independently predicted poor outcome together with female sex (OR 1.5; CI 1.1–2.1), higher age (65–74: OR 1.7; CI 1.1–2.8, 75–84 years: OR 3.3; CI 2.1–5.3; ≥85 years OR 14.5; CI 7.4–28.5), higher NIHSS on admission (OR 1.2; CI 1.1–1.2), diabetes mellitus (OR 1.8; CI 1.3–2.5), probable atherothrombotic stroke (OR 1.8; CI 1.1–2.8) mRS > 2 at hospital discharge (OR 8.9; CI 6.0–13.0) and leukocyte count of > 9.000/mm3 (OR 1.4; CI 1.0–1.9);(Table 2, Model A). Threshold that was reported in previous studies [48]. Exclusion of patients who died < 7 days after stroke (Model B) resulted in comparable results.

**Table 2:** Results of multivariate logistic regression analysis on 1-year functional outcome after first-ever ischemic stroke. Model A (all observations), Model B (excluding patients that had died early within 0–7 days after hospital admission) and Model C (all observations with subanalysis of the first-ever ischemic stroke “Unknown cause”). Observations with one or more missing variables have been dropped from multivariate analysis. Bold values indicate significance at *p* <  0.05. Adjusted for age, diabetes mellitus and stroke severity

Predictor variable	Model A	*n* = 1234	Model B	*n* = 1230	Model C	*n* = 1234
	OR (95% CI)	*p* value	OR (95% CI)	*p* value	OR (95% CI)	*p* value
Sex						
*(W* vs *M)*	1.5 (1.1–2.1)	0.01	1.5 (1.1–2.1)	0.01	1.5 (1.1–2.1)	0.01
Age, years *(*vs *55–64)*						
< 55	0.4 (0.2–0.8)	0.01	0.3 (0.2–0.8)	0.01	0.4 (0.2–0.8)	0.01
65–74	1.7 (1.1–2.8)	0.03	1.7 (1.1–2.8)	0.03	1.7 (1.0–2.8)	0.03
75–84	3.3 (2.1–5.3)	< 0.01	3.3 (2.1–5.3)	< 0.01	3.3 (2.0–5.3)	< 0.01
≥ 85	14.5 (7.4–28.5)	< 0.01	14.5 (7.4–28.5)	< 0.01	14.4 (7.4–28.3)	< 0.01
Regular Physical Activity						
*(No* vs *Yes)*	1.7 (1.1–2.5)	< 0.01	1.7 (1.1–2.5)	< 0.01	1.7 (1.1–2.5)	< 0.01
Diabetes						
*(Yes* vs *No)*	1.8 (1.3–2.5)	< 0.01	1.9 (1.3–2.95	< 0.01	1.8 (1.3–2.5)	< 0.01
Leukocyte count						
*(≥ 9* vs *< 9 × 1000/mm*^*3*^*)*	1.4 (1.0–1.9)	0.04	1.4 (1.0–1.9)	0.05	1.4 (1.0–1.9)	0.04
TOAST						
*(*vs *Small-artery occlusion)*						
Probable atherothrombotic	1.8 (1.1–2.8)	0.02	1.8 (1.0–2.8)	0.02	1.8 (1.1–2.8)	0.02
Cardioembolic	1.4 (0.9–2.1)	0.12	1.4 (0.9–2.1)	0.13	1.4 (0.9–2.1)	0.12
Large-arthery Atherosclerosis	1.4 (0.9–2.3)	0.17	1.4 (0.9–2.3)	0.17	1.4 (0.9–2.3)	0.17
Other determined	2.0 (0.9–4.4)	0.09	2.0 (0.9–4.4)	0.09	2.0 (0.9–4.4)	0.09
Unkown	1.5 (0.8–3.0)	0.21	1.5 (0.8–3.0)	0.21		
Cryptogenic ESUS					2.2 (0.9–5.5)	0.10
Cryptogenic NON-ESUS					1.3 (0.5–3.7)	0.62
Undetermined source					0.9 (0.2–3.8)	0.94
mRS at hospital discharge						
*(≥ 3* vs *< 3)*	8.9 (6.0–13.3)	< 0.01	8.9 (6.0–13.3)	< 0.01	8.9 (6.0–13.4)	< 0.01
NIHSS on admission						
*(per point)*	1.2 (1.1–1.2)	< 0.01	1.2 (1.1–1.2)	< 0.01	1.2 (1.1–1.2)	< 0.01

*ESUS* embolic stroke of unknown source, *mRS* modified Rankin Scale, *NON-ESUS* no embolic stroke of unknown source, *N* number, *NIHSS* National Institutes of Health Stroke Scale, *SD* standard deviation, *TOAST* Trial of ORG 10172 in Acute Stroke Treatment;

Subclassifying patients with unknown stroke etiology the diagnosis of cryptogenic ESUS showed a trend towards loss of functional outcome or death (OR 2.2; CI 0.9–5.5) whereas cryptogenic Non-ESUS stroke did not (OR 1.3; CI 0.5–3.7) (Table 2, Model C).

## Discussion

In addition to well known predictors of stroke outcome we identified lack of regular PA prior to stroke, high leukocyte count, probable atherothrombotic stroke etiology as independent predictors of poor functional outcome. Stroke severity (measured by NIHSS or mRS), diabetes mellitus and higher age are well established independent predictors for poor stroke outcome [7, 49–53]. Recently published scoring systems also used parameters like arterial hypertension, AF, higher age, sex, blood glucose, level of consciousness, stroke type or severity to predict stroke outcome [3, 7, 54, 55]. Factors like arterial hypertension, AF, CAD, hypercholesterolemia, PAD, smoking status and alcohol consumption were not independent predictors for loss of functional independence in our population. This may partly be explained by too low numbers of subjects in our study, resulting in insufficient statistical power to detect predictors with moderate impact. Moreover, differences between study populations in risk factor control and compliance to medication intake (e.g. oral anticoagulants in AF) may be accountable for variations between studies.

Lack of regular physical activity is a modifiable risk factor for both, ischemic and hemorrhage stroke [56–58]. PA reduces stroke risk by lowering blood pressure, improving lipid and glucose metabolism and endothelial function. Further benefits are reduction of thrombocyte aggregation and blood viscosity [58]. Recently published studies showed endothelial function and atherogenesis to be influenced by PA [59, 60]. Improved physical fitness results in better control of risk factors like hypertension and diabetes mellitus and this could have contributed to the beneficial effect of PA on stroke outcome in our study. Patients who had engaged in regular PA may be in better physical and mental conditions and have more capacity to cope with the sequelae of stroke. An association between prognosis after stroke and previous PA had been reported in few studies so far [56–58]. In the Framingham study there was no reduction for stroke risk beyond a moderate level of physical activity [26]. We therefore used comparable low threshold (≥1 per week) to define regular PA in our study. Physical handicap before stroke was seldom observed (mRS > 2 in only 4.9% of patients) in our patients and did thus, not explain the association between lack of PA and outcome. A strength of our study is higher number of cases and prospective study design compared to other studies. However, our findings should be ascertained by further prospective studies. PA is a modifiable lifestyle risk factor with a major importance in preemptive strategies to prevent strokes and improve mid-term stroke outcome. For future stroke care, PA as a modifiable risk factor should get more focus in stroke research as well as in primary and secondary stroke care. Documenting and focusing on regular PA might have great impact in primary stroke care leading to better stroke outcomes and more functional independence. Being physically active in mid-life increases the odds of being active in old age [61]. However, more research is necessary, e.g. to find out how much PA preceeding first-ever ischemic stroke reduces stroke severity. Visual representation of the overall distribution of mRS 12 months post stroke draws a clear picture of difference by PA activity pre stroke. Even if a part of the difference is attributable to other confounding factors, like NIHSS on admission and mRS at hospital discharge, the cumulative barchart visualizes striking difference of outcomes in favour of PA. In contrast to other studies, we used definition of the German Olympic Sports Association. Registration of leisure-time motor activity only might result in underestimation of physical activity.

Higher leukocyte count was associated with lack of functional independence in our population indicating that the strength of the early inflammatory response heralds poor prognosis independent of clinical stroke severity and factors that are known to contribute to leukocyte counts such as smoking and diabetes mellitus. Infection before ischemia is an established stroke trigger factor which may partly explain the association between leukocyte count and poor prognosis, as well as larger infarct volume which was not investigated in our study [31, 32]. Blood samples were taken within 24 h in the majority of patients; therefore it is unlikely that leukocytosis was due to stroke related infections such as post-stroke pneumonia.

In line with other studies, female sex proved to be an independent predictor for lack of functional independence [7, 62]. Gall et al. hypothesized that females are more vulnerable than men because of differences in chronic diseases, socioeconomic status and medical histories [63]. Females are more likely to suffer from severe strokes [62, 64]. A further reason might be the sex-related differences in muscular strength, or different approaches to handle their disabilities. This difference between sexes may increase in the elderly, because the observed decline in muscle strength with aging is also related to a reduction in PA, normally different between sexes. In people older than 65 years, less than one third of all women performed some PA - compared to men with 47.9%. Elderly women with a higher body mass index have a lower status of PA [65]. Moreover, female patients have a higher risk of walking with a cane [66]. More focus on PA in middle-life could improve level of PA in elderly people [61]. Additionally, more frequent occurrence of depression and lack of social support may increase probability for loss of functional independence in females [7, 55, 67]. However, most authors used univariate analyses and did not adjust for confounders.

Regarding stroke etiology, probable atherothrombotic stroke was an independent predictor for loss of functional independence in our population. The category of stroke of probable atherothrombotic etiology includes patients with distinct signs of non-stenosing arteriosclerosis as a marker of probable atherosclerotic plaques mostly at the orifices of small penetrating arteries, such as lenticulostriate arteries. CAD and PAD, that were associated with loss of functional independence in univariate but not in multivariate analysis are other common atherosclerotic diseases and may have contributed to some degree to the effect of this etiologic subgroup. This group may also include some patients with cardioembolic strokes due to non-detected AF. In contrast to other studies, cardioembolism was not independently associated with poor functional outcome in multivariate analysis. Cardioembolic stroke results in higher stroke severity. Adjustment for NIHSS and mRS at discharge presumeably prompted lack of significance of cardioembolism in our study.

In 117 patients stroke etiology was classified as stroke of unknown origin. Further analysis identified cryptogenic strokes and ESUS as main contributors to this group. Two thirds of patients with cryptogenic strokes were diagnosed as cryptogenic ESUS and tended to be associated with loss of functional independence, a finding that did not reach statistical significance due to small numbers. These cases may partly represent patients with non-detected AF and without any kind of anticoagulation. More effort in detecting AF in patients with ESUS may reduce recurrence of IS and improve functional outcome.

There are strengths and limitations to this study. The data were derived from a population–based stroke register, including both hospitalized and non-hospitalized patients without age-restrictions. Data have been collected prospectively by applying standardized protocols using multiple notification sources widely ensuring complete case ascertainment. Robust quality of case ascertainment is indicated by stable incidence rates over time. A high rate of neuroimaging (98.2%) assures high reliability of first-ever ischemic stroke diagnosis. An intense clinical work-up together with the application of modified TOAST criteria resulted in a low number of patients with undefined stroke causes [48]. This enabled us to study a relatively large cohort of unselected patients, resulting in sufficient statistical power to determine differences in stroke outcome. Our study is characterized by a high rate of follow-up and low rates of missing values (< 5%). Observed early death rates in our study were similar to findings in other population-based stroke registers [36, 68].

Limitations of our current work include the lack of data on the quality of risk factor control and the exploratory character of our results on factors like previous PA without possibility to reanalyze the detected results in a derivation sample. Further statistical limitation is lack of a power as LuSSt is primarily descriptive. Additionally, PA after stroke has not been assessed and an overestimation in amount of sports activity might result in another bias: acquisition of self-reported patients data increase the possibility of a misclassification bias. Distant events are often not precisely recalled, also raising the possibility of recall bias, particularly for sports in early adulthood. In contrast to other studies on physical activity using WHO definition, we used definition of the German Olympic Sports Association. Furthermore, poor outcome had not included standardized patient reported outcome measures like Euro-QOL-5, Frenchay Activities Index, HAD’s depression or PROMIS-10 [55, 69–71].

## Conclusion

In addition to broadly accepted risk factors for poor functional outcome we found that lack of PA prior to stroke, high leukocyte count at admission and probable atherothrombotic stroke etiology may constitute important independent predictors of loss of functional independence after first-ever ischemic stroke.

## Abbreviations

AF: Atrial fibrillation
CAD: Coronary artery disease
CI: Confidence interval
CRP: C-Reactive Protein
ESUS: Embolic stroke of unknown source
ICH: Intracerebral hemorrhage
IS: Ischemic stroke
LuSSt: Ludwigshafen Stroke Study
mRS: Modified Rankin Scale
NIHSS: National Institute of Health Stroke Scale
OR: Odds ratio
PA: Physical activity
PAD: Peripheral artery disease
rt-PA: Recombined tissue plasmin activator
SAH: Subarachnoid hemorrhage
TIA: Transient ischemic attack
WHO: World Health Organization
